# Retraction: Exosomes Mediate LTB_4_ Release during Neutrophil Chemotaxis

**DOI:** 10.1371/journal.pbio.3001320

**Published:** 2021-07-07

**Authors:** Ritankar Majumdar, Aidin Tavakoli Tameh, Carole A. Parent

After this article [1] was published, the corresponding author noted concerns about some of the reported results. In response, the National Institutes of Health (NIH) conducted an investigation and concluded that there was evidence of data fabrication and/or falsification in Figs 2 and 3.

Specifically:

- Compared to the primary data, Figs 2A, 2B, 2C, 2G and 3H report images in which dots representing 5-lipoxygenase (5-LO) immunogold signal were added and removed in a manner that supported the article’s hypothesis.
- Immunogold quantification data in Fig 2F do not provide a true representation of the experimental results.
- Cell organelle membranes and/or subcellular vesicles were selectively added and removed in Fig 2G.
- In Fig 2A and 2D, two images obtained from the same experimental sample are reported as representing different experimental results.
- The scale bars in Figs 2H, 3D, 3H are not correct, and the quantitative measurement data shown in the graph in Fig 3D do not accurately represent vesicle sizes as per the raw data. The corresponding author confirmed that the scale bars were reported incorrectly in the indicated panels: they should have read 800 nm in panel 2Hii, 400 nm in panel 2Hiii, and 100 nm in Fig 3D.
- In the raw image data for Fig 3H, only one exosome in a field of >30 was associated with CD63 immunogold labelling. As such, the figure legend description of this as “representative” of the overall results is not accurate. The corresponding author agreed with this but commented that the claim about the presence of CD63 on exosomes was supported by other results reported in Fig 3C, Fig 3F, and Fig 5A.

In addition, the NIH advised that questions were raised about the 5-LO blot in Fig 1C, and that per their assessment of the primary records, this issue was not fully resolved and the blot published in [1] is unreliable. The corresponding author commented that Fig 1C reports the correct findings and provided PLOS evidence from a replicate experiment that lends support for the results.

Following review by an investigation committee, NIH concluded that the above issues resulted from misconduct by the first author; no other authors were found to be implicated.

The authors retract the article due to the concerns confirmed by the investigating committee about the integrity and reliability of the aforementioned results. All authors agree with this retraction. The first author, while agreeing with retraction, disputes the NIH’s finding of misconduct.

The corresponding author apologizes for the issues with the article and stands by the validity and reliability of the article’s major findings. A corrected version of this article with the problematic electron microscopy data removed and new data presented for Fig 1C and Fig 3D (now 2D) has been peer reviewed by *PLOS Biology* and is being published [2] coincident with this retraction.
